# Complications after thermal ablation for thyroid nodules across countries

**DOI:** 10.3389/fendo.2025.1608164

**Published:** 2025-07-29

**Authors:** Shuang Wu, Yantao Cai, Xiaoyu Liu, Chenfang Zhu

**Affiliations:** ^1^ Department of General Surgery, Shanghai Ninth People’s Hospital, Shanghai Jiao Tong University School of Medicine, Shanghai, China; ^2^ Department of Molecular Diagnostics & Endocrinology, The Core Laboratory in Medical Center of Clinical Research, State Key Laboratory of Medical Genomics, Shanghai Ninth People’s Hospital Affiliated to Shanghai Jiao Tong University School of Medicine, Shanghai, China

**Keywords:** ablation techniques, complication, thyroid nodule, papillary thyroid microcarcinoma, incidence

## Abstract

**Objective:**

Thermal ablation is an effective treatment for thyroid nodules; however, the range of complications associated with thermal ablation is unclear. In this study, we analyzed the complications of thyroid nodule thermal ablation in our hospital in combination with data from different countries for further analysis to determine the complications.

**Methods:**

536 patients treated by thermal ablation at Shanghai Ninth People’s Hospital from Jan 2021 to Dec 2023 were enrolled in this retrospective study. The types and numbers of complications were recorded. Studies reporting complications of thermal ablation for thyroid nodules were identified in the PubMed database from Jan 2004 to Mar 2024. The incidence rate and types of complications were analyzed. Finally, we compared our data with those studies.

**Results:**

Twelve types of complications associated with thermal ablation occurred to patients in our hospital. The overall incidence was 10.82%. The top five complications were pain, edema, voice changes, hematoma, and vascular reactions. In addition, 21 kinds of complications were reported across 13 countries, and the average incidence rate was 12.61%. The top five complications were pain, vocal changes, edema, hematoma, and fever. The total incidence rate did not significantly differ between our hospital and other countries (P ≥ 0.05), though the incidences of specific complications, including tracheal injury, vasovagal reaction, dyspnea, scarring, infection and fever, were significantly different (P < 0.05).

**Conclusions:**

The incidence of complications associated with thermal ablation of thyroid nodules is similar across countries. However, the proportions and types of complications vary regionally.

## 1 Introduction

The thermal ablation (TA) procedure, proposed as a minimally invasive treatment method, has received increasing attention in recent years (1–4). TA methods include radiofrequency ablation (RFA), microwave ablation (MWA), laser ablation (LA), and high-frequency focused ultrasound (HIFU) (5). The principle of these methods involves the induction of tissue coagulation necrosis under extreme hyperthermic conditions (6). TA is a suitable treatment for benign thyroid nodules with ≥ 10% solid components (7). Compared with surgery, the advantages of thyroid TA include safety, efficacy, low cost, good tolerability and preservation of thyroid function (8, 9). The 2022 international multidisciplinary consensus explicitly stated that TA can be considered for symptomatic thyroid nodules (6). Furthermore, multiple other global follow-up studies have confirmed the effectiveness of TA for benign nodules and papillary thyroid microcarcinoma (PTMC) (10–14); however, the status of complications of TA is unclear. In this study, data regarding the complications of thyroid TA at our hospital were combined with those in different countries to analyze the range of complications associated with thyroid TA.

## 2 Materials and methods

The study was approved by the Ethics Committee of Shanghai Ninth People’s Hospital, Shanghai Jiao Tong University School of Medicine (SH9H-2021-MT6-1) and informed consent for each procedure was obtained from all patients prior to the procedure.

### 2.1 Study population

A total of 536 patients who underwent thyroid MWA at our hospital (SHNH) from Jan 2021 to Dec 2023 were retrospectively enrolled in this study. All of the patients underwent ultrasound (US) prior to TA to record the tumor number, location, and volume, and fine-needle aspiration (FNA) was performed before TA to confirm the pathology. The clinical characteristics, volume of tumor change before and after TA, and complications associated with TA were recorded. The inclusion criteria included patients who were clearly diagnosed with symptomatic benign thyroid nodules (Bethesda II or III, diameter ≥ 25 mm) or low-risk PTMCs (diameter ≤ 10 mm) by FNA and who were treated with MWA. Complications were recorded immediately, at 3 months and at 6 months after MWA. The exclusion criteria were as follows: (1) Patients who had other types of thyroid cancer; (2) Patients who were confirmed to have central lymph node metastasis or lateral lymph node metastasis before the operation; (3) Patients who had a history of thyroid operations. Informed consent was obtained from all patients in this study. This retrospective study was approved by the institutional review board.

### 2.2 Surgical procedures

All patients underwent ultrasound to confirm the size and location of their thyroid nodules prior to TA. The patients lay flat on their back with a soft pillow under their shoulders and with their neck tilted backward. After disinfection, 1% lidocaine was used for local anesthesia and fluid isolation. Moving TA was used for large benign thyroid nodules, and fixing TA was used for PTMCs via 30 W MWA (Great Wall MTI-5A, China; XR-1610, China).

### 2.3 Literature search

**Figure 1:**
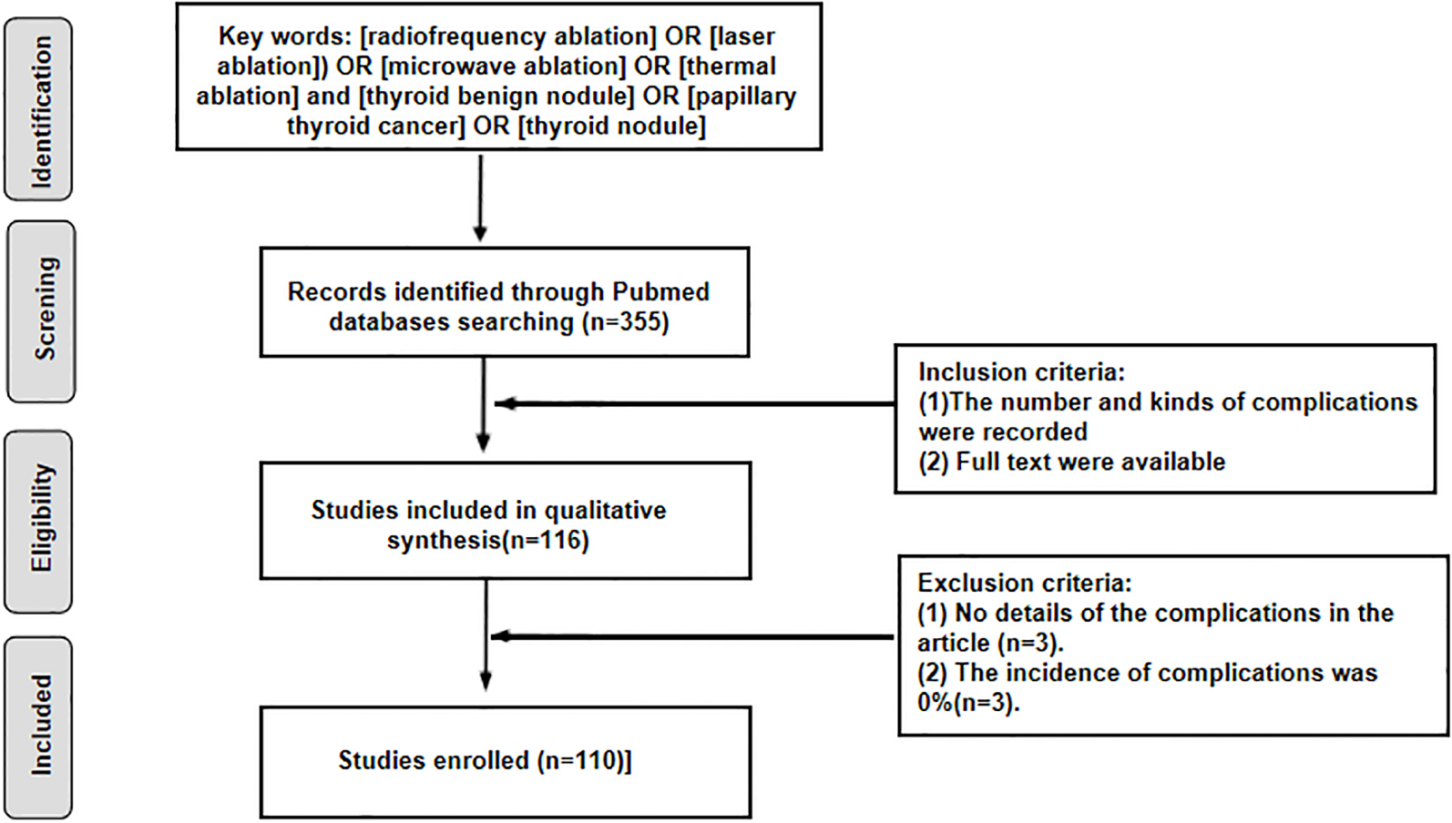
Study flow diagram.

The PubMed database was searched for original literature regarding complications of thyroid TA from 2014–2024. Research reported in English was included in this study. The flowchart for article filtering is shown in **Figure 1**. The inclusion criteria were as follows: (1) patients with benign thyroid nodules or PTMCs ≤1 cm who underwent RFA, MWA, LA or HFU. (2) The incidence and types of complications of thyroid TA were reported. The exclusion criteria were as follows: (1) The details of the complications were not reported in the publication. (2) The incidence rate of TA was 0%. The included studies were classified and analyzed according to the country of origin.

### 2.4 Statistical analysis

In this study, continuous variables are presented as the means ± standard deviations (SDs). Categorical data are presented as numbers (%). Comparisons between the complication rates at SHNH and those in the reported studies were performed via the chi-square test. P < 0.05 was considered to indicate statistical significance. All the statistical analyses were conducted via R (version 4.3.1).

## 3 Results

### 3.1 Data analysis in SHNH

A total of 536 patients were included in this study, which included 418 females (78.0%) and 118 males (22.0%). The patients ranged in age from 16 to 81 years (48.49 ± 14.85 years). The numbers of thyroid nodules on the left side, right side and isthmus were 250 (46.6%), 275 (51.3%) and 11 (2.1%), respectively. The average volume of thyroid nodules before TA and 3 months after TA ranged from 17281.85 ± 25960.74 mm³ to 5531.97 ± 16482.36 mm³ (P < 0.05). There were 431 benign thyroid nodules and 105 PTMCs. A total of 78 patients with PTMC were less than 55 years old, and 27 patients with PTMC were 55 years old or older. A total of 12 complications were recorded, and the incidence of TA was 10.82%. The top five complications were pain (5.25%), edema (1.26%), voice changes (1.26%), hematoma (1.05%), and vascular reactions (1.05%) (**Table 1**). Only one patient experienced Horner’s syndrome, tracheal injury or cough (**Table 1**).

**Table 1:** Different types of complications of thyroid TA in patients at SHNH.

Complications	SHNH n=536 (%)
Pain	25 (5.23)
Horner’s syndrome	1 (0.21)
Tracheal injury	1 (0.21)
Cough	1 (0.21)
Dyspnea	2 (0.42)
Skin burn	2 (0.42)
Scar	2 (0.42)
Infection	2 (0.42)
Vasovagal reaction	5 (1.05)
Hematoma	5 (1.05)
Voice change	6 (1.26)
Edema	6 (1.26)
Total	58 (10.82%)

### 3.2 Related literature

A total of 110 studies involving a total of 14,668 patients from 13 countries were identified in the literature search. Sixty-nine studies employed RFA, 26 studies employed MWA, 17 studies employed LA, 5 studies employed HIFU, and three studies did not report the treatment methods. There was only one study each from Bulgaria and Singapore.

**Figure 2:**
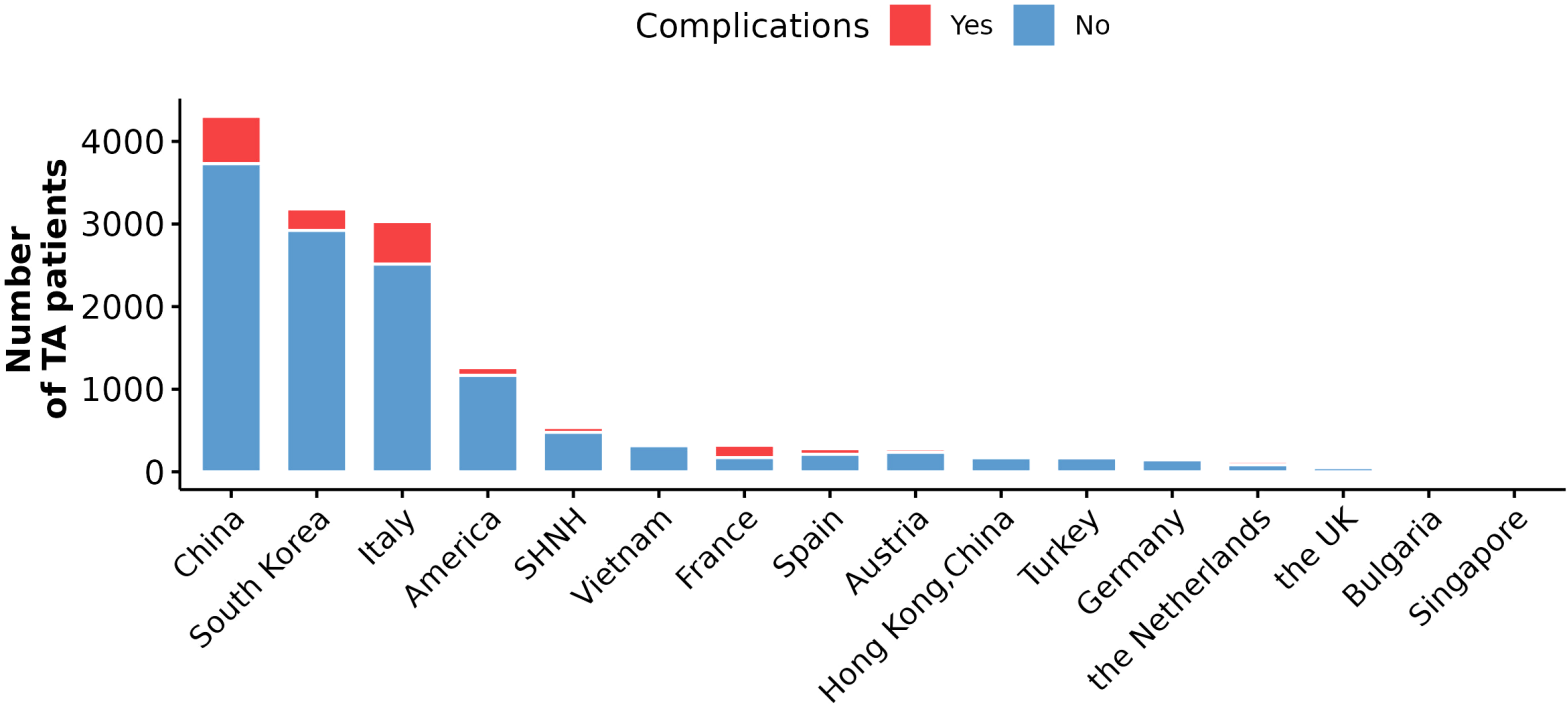
The number of TA patients and complications in different countries. Red bar indicates the number of TA patients with complications, and blue indicates the number of TA patients without any complications included in this study.

The five countries with the greatest number of patients with TA were China (5481), South Korea (3182), Italy (3029), the United States (1261) and Vietnam (341). The lowest number of cases were reported in the Netherlands (124), Bulgaria (20) and Singapore (10). The countries with the highest number of complications of TA were China (626, 11.42%), South Korea (260, 8.17%), Italy (515, 17.00%), the United States (94, 7.45%) and Vietnam (25, 7.33%) (**Figure 2**).

**Figure 3:**
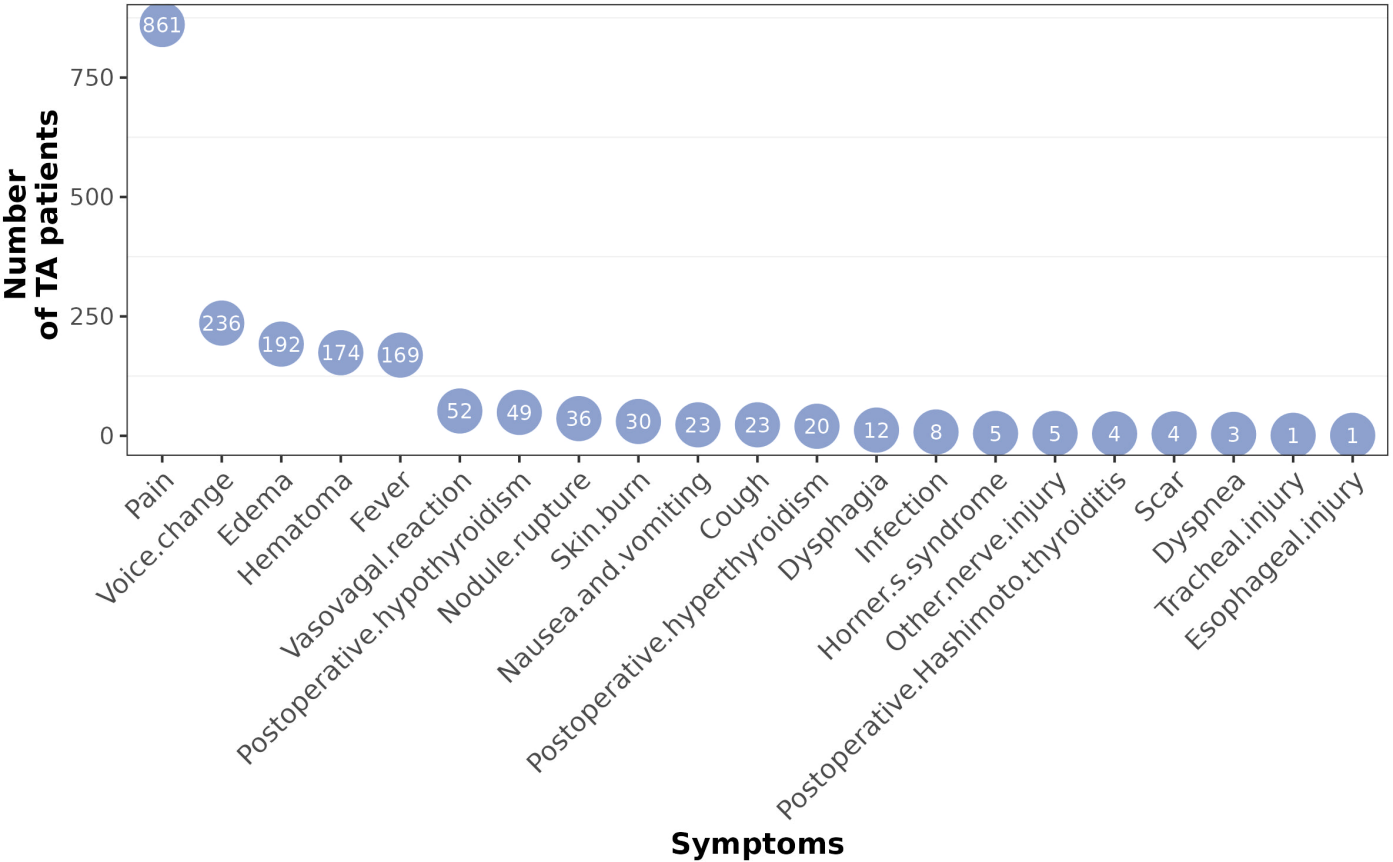
Number of different complications after thyroid TA. All countries were summarized together.

A total of 21 complications were reported. The top five complications were pain (861), voice changes (236), edema (192), hematoma (174), and fever (169). Pain was the most common complication, and the number of pain cases was more than three times greater than the number of voice changes and four to ten times greater than the number of other complications. Other complications included vasovagal reactions, postoperative hypothyroidism, nodule rupture, skin burn, nausea and vomiting, cough, postoperative hyperthyroidism, dysphagia, infection, Horner’s syndrome, other nerve injury, scarring, dyspnea, tracheal injury, and esophageal injury. Tracheal injury and esophageal injury were rare complications, with only one case reported (**Figure 3**).

**Figure 4:**
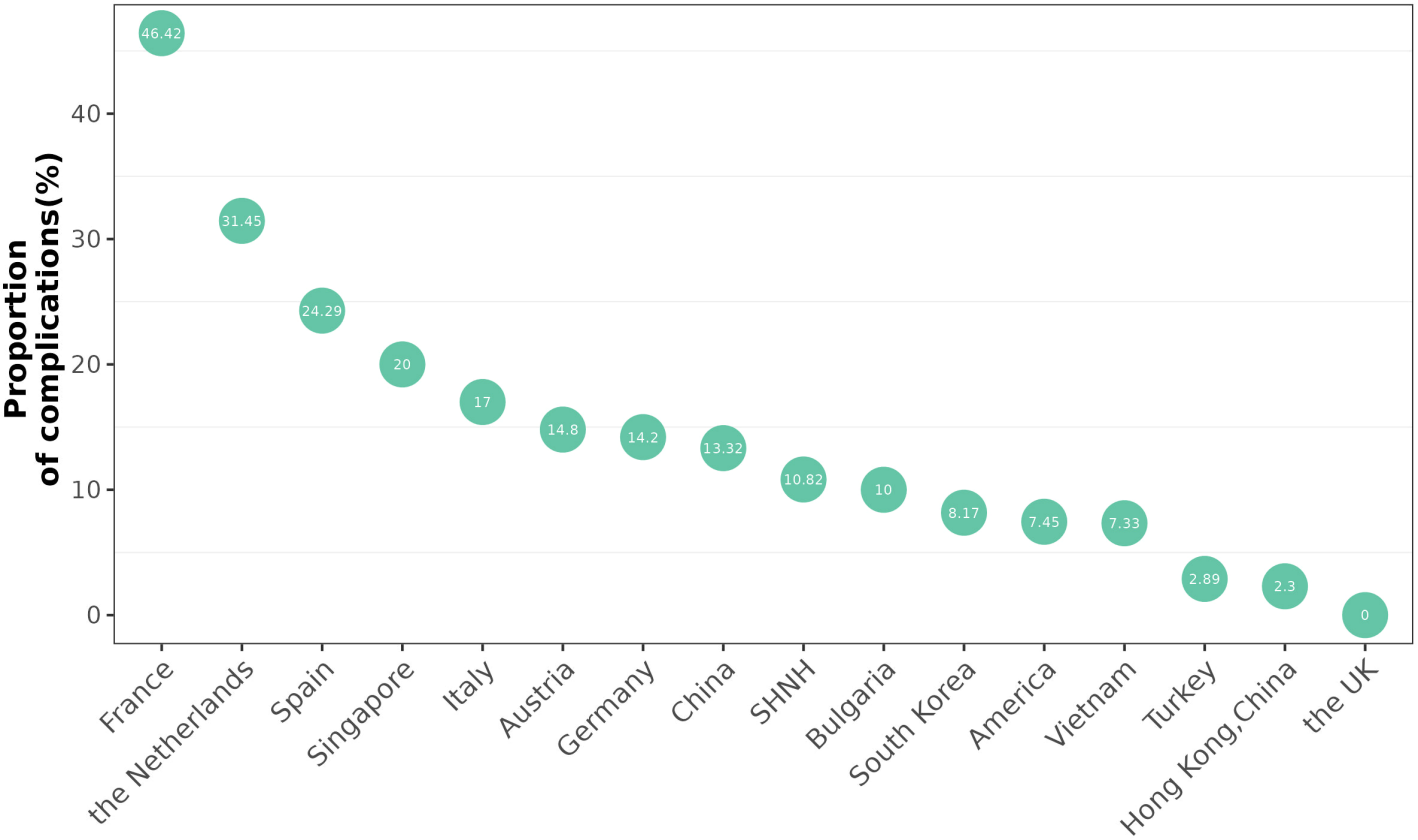
Proportion of complications in different countries.

The five countries with the highest incidence of complications were France (46.62%), the Netherlands (31.45%), Spain (24.29%), Singapore (20%) and Italy (17%). The incidence of complications was lowest in Turkey (2.89%). The incidence of complications in China was 11.42% (**Figure 4**).

**Figure 5:**
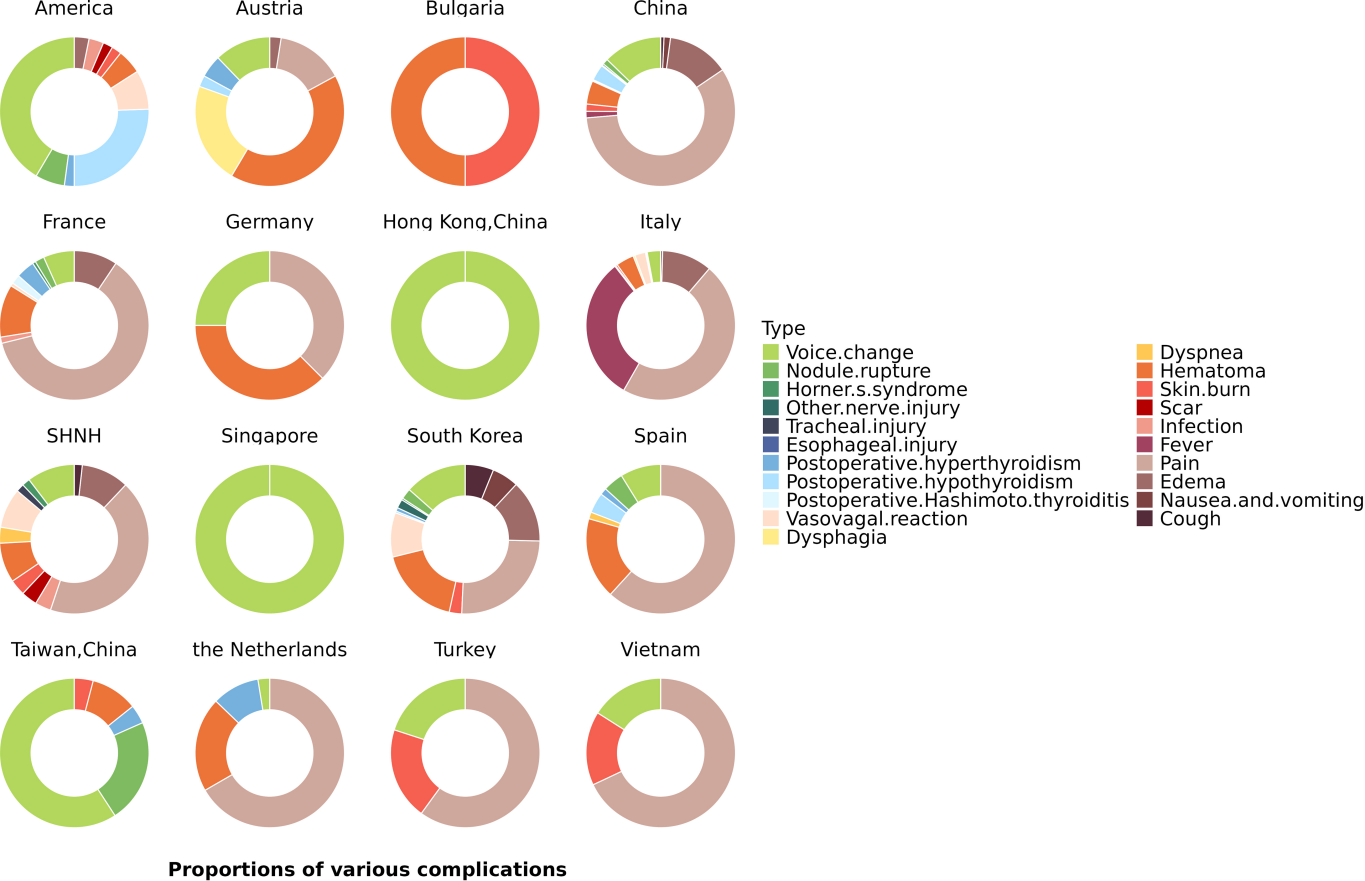
Different proportions of various complications in different countries. Pie charts showing the proportions of complications in each country.

The proportions of various complications in different countries are shown in **Figure 5**. Voice change and postoperative hypothyroidism were the most common complications in the United States, and hematoma and dyspnea were the most common complications in Australia. Pain and fever were the most common complications in Italy. The composition of complications was similar in China and Korea (**Figure 5**).

There was no significant difference in the incidence of TA complications between SHNH (10.82%) and institutions in China (11.42%), though the incidences of vasovagal reactions (1.05%), dyspnea (0.42%), scarring (0.42%) and infection (0.42%) significantly differed between the two locations (P < 0.05). When the complications of thyroid TA in patients treated at SHNH and those treated in different countries were compared, the incidences of tracheal injury, vasovagal reactions, dyspnea, scarring, infection and fever were significantly different between SHNH patients and those in different countries (P < 0.05). However, there was no significant difference in the total incidence of complications at SHNH and in different countries (**Table 2**).

**Table 2:** Comparison of complication morbidity between SHNH patients and those in different countries.

Complications	SHNH: n=536 (%)	Other countries: n=14668 (%)	*P*
Tracheal injury	1 (0.21)	0 (0.00)	0.0360*
Vasovagal reaction	5 (1.05)	47 (0.37)	0.0383*
Dyspnea	2 (0.42)	1 (0.01)	0.0038*
Scar	2 (0.42)	2 (0.02)	0.0003*
Infection	2 (0.42)	6 (0.05)	0.0213*
Fever	0 (0.00)	169 (1.32)	0.0031*
Total	58 (10.82)	1850 (12.61)	1.0000

*Statistical significance was set at *P* < 0.05.

## 4 Discussion

TA is widely used, though the indications for TA remain unclear. The existing guidelines are mostly for RFA and can be used for the treatment of benign thyroid nodules, PTMCs, and recurrent thyroid cancers (RTCs). Thermal ablation of thyroid cancer is only indicated for single low-risk PTMCs, the low degree of invasiveness should be verified prior to ablation. For patients with RTC, curative-intent RFA should be considered when complete removal of the tumor is possible in patients with RTC and a limited number (≤ 3) of small tumors (size ≤ 2 cm). RFA can be performed with curative or palliative intent for RTCs at the thyroidectomy bed, neck dissection site, or metastatic cervical lymph nodes for patients who refuse surgery or are at high surgical risk (15, 16).

Some studies have reported the use of RFA for other types of PTC, including PTC larger than 1 cm, and palliative treatment for recurrent and metastatic PTC. The results are encouraging and provide a safe and effective treatment option for patients with PTC who cannot undergo or refuse surgery. However, further research is needed (17).

The management of indeterminate nodules with TA is not recommended as it is difficult to achieve diagnostic needs and TA cannot prevent metastasis. In the 2023 Bethesda System for Reporting Thyroid Cytopathology, Bethesda category III is simplified as atypia of undetermined significance (AUS), which is subdivided into “AUS nuclear” and “AUS other” (18). AUSs with nuclear atypia have significantly greater ROMs and require more cautious clinical management, corresponding to research results since the publication of the second edition of the classification (19–21).

In the current study, all patients who received TA treatment at SHNH underwent biopsy to confirm the pathological diagnosis of benign nodule or PTMC, with no history of lymph node metastasis or previous thyroid surgery. TA was used as a minimally invasive initial treatment to alleviate patient pain, reduce costs, and improve safety and aesthetics. However, the probability of serious postoperative complications, such as tracheal injury, remains. Therefore, the indications for TA must be further clarified. Moreover, considering that the probability of serious complications still occurs, strict inclusion criteria for TAs are very important.

There are several reports and reviews involving TA complications. The 2022 international multidisciplinary consensus concluded that the two most common postoperative complications are recurrent laryngeal nerve injury and thyroid nodule rupture (6). These two situations can be separately treated by injection of a cold irrigant into the region of suspected thermal injury (22) and by conservative management (23). A meta-analysis of the incidence of common complications was conducted (24) and reported values of 1.0%-2.0%, 0.2%-0.5%, 1.0%-100%, 0.0%-17.0%, and 0.27%-3.7% for nerve injury, nodule rupture, pain, hematoma, and skin burns, respectively. However, comparisons across countries are lacking. In the present study, we compared the complications of thyroid TA across different countries with our own data for further statistical analysis. We found that TAs have been widely applied in China (25, 26), South Korea (27, 28) and Italy (29, 30). Common complications include pain, vocal changes, edema, hematoma, fever, etc. (31–33). Uncommon complications are tracheal injury and esophageal injury. Fu Q reported in 2021 that esophageal injury, which was located in the esophageal serosa, was caused by insufficient observation during the procedure. The patient experienced foreign body sensations while swallowing after ablation and recovered one month after the ablation procedure (34). Two case reports were analysed in the previously published literature. Patients experienced intense pain and difficulty breathing one to two weeks after the ablation procedure; possible contributing factors were an excessive thyroid nodule volume, a prolonged ablation time, and deep sedation anesthesia (35, 36). We also reported a case of airway fistula after MWA. The patient developed neck pain, fever, cough and increased sputum after surgery. CT revealed small air bubbles on the right side of the airway with inflammation of the neck tissue. The patient’s symptoms disappeared one month after anti-infective treatment.

Our study is the first to compare complications regionally. This retrospective study revealed that the region with the highest incidence rate of complications after ablation was Europe, including France (14, 37, 38) and the Netherlands (32, 39). France has the highest incidence rate of complications (46.42%). A total of 321 patients were included in three studies conducted in France; the incidence rates of TA complications were 15.6% (14), 6.2% (37) and 78.9% (38). The incidence in the Netherlands ranks second (21.45%). There are two reasons for the high incidence rates of TA complications in France and the Netherlands. One reason is that the number of thyroid TA procedures in these two countries was limited (France reported 321 cases, and the Netherlands reported 124 cases), and the proficiency of the operator was thus affected. The other reason is that most of the complications reported in France and the Netherlands involved pain, and this factor increased the proportion of complications. For example, Ben Hamou reported that 92 out of 166 patients experienced pain, leading to an incidence of up to 78.9% (39). In some countries, pain was not included in the reported complications.

In addition, we compared the complication data of various countries in the previous literature with those of our single centre, SHNH. There are significant differences in the incidence of certain single complications, but there is no significant difference in the incidence of overall complications.

This study has several limitations. The determination of some complications is subjective, which affects the results involving pain. In addition, the follow-up time of previous studies varied from six months to five years, and most of them were retrospective studies. Furthermore, there are very few cases reported in some countries, such as Singapore and Bulgaria. Further attention and research are needed in this area.

## 5 Conclusion

The incidence rate of complications associated with thyroid TA is approximately 10% to 15%. However, the proportions and types of complications vary reionally.

## Data Availability

The original contributions presented in the study are included in the article/**Supplementary Material**, further inquiries can be directed to the corresponding author/s.
