# Supplementary material for: Complications after thermal ablation for thyroid nodules across countries
Source: Front Endocrinol (Lausanne). 2025 Jul 29;16:1608164. doi: 10.3389/fendo.2025.1608164 (PMC12339322; doi:10.3389/fendo.2025.1608164)
Supplement: Supplementary file 1 [file DataSheet1.docx]

Supplementary Material

# Literature enrolled in this study

[1]Lee M K, Lee S W. Analysis of 5 years' experience of a head and neck surgeon with radiofrequency ablation for benign thyroid nodule[J]. American Journal of Otolaryngology, 2023, 44(2): 103715.

[2]Jeong S Y, Ha E J, Baek J H, et al. Assessment of thyroid-specific quality of life in patients with benign symptomatic thyroid nodules treated with radiofrequency or ethanol ablation: a prospective multicenter study[J]. Ultrasonography, 2022, 41(1): 204.

[3]Cho S J, Baek S M, Lim H K, et al. Long-term follow-up results of ultrasound-guided radiofrequency ablation for low-risk papillary thyroid microcarcinoma: more than 5-year follow-up for 84 tumors[J]. Thyroid, 2020, 30(12): 1745-1751.

[4]Di Q. RE: efficacy and safety of radiofrequency ablation for benign thyroid nodules: a prospective multicenter study[J]. Korean journal of radiology, 2018, 19(3): 542.

[5]Ha S M, Sung J Y, Baek J H, et al. Radiofrequency ablation of small follicular neoplasms: initial clinical outcomes[J]. International Journal of Hyperthermia, 2017, 33(8): 931-937.

[6]Kim C, Lee J H, Choi Y J, et al. Complications encountered in ultrasonography-guided radiofrequency ablation of benign thyroid nodules and recurrent thyroid cancers[J]. European radiology, 2017, 27: 3128-3137.

[7]Baek J H, Ha E J, Choi Y J, et al. Radiofrequency versus ethanol ablation for treating predominantly cystic thyroid nodules: a randomized clinical trial[J]. Korean journal of radiology, 2015, 16(6): 1332-1340.

[8]Lim H K, Lee J H, Ha E J, et al. Radiofrequency ablation of benign non-functioning thyroid nodules: 4-year follow-up results for 111 patients[J]. European radiology, 2013, 23(4): 1044-1049.

[9]Baek J H, Lee J H, Sung J Y, et al. Complications encountered in the treatment of benign thyroid nodules with US-guided radiofrequency ablation: a multicenter study[J]. Radiology, 2012, 262(1): 335-342.

[10]Russ G, Ben Hamou A, Poirée S, et al. Learning curve for radiofrequency ablation of benign thyroid nodules[J]. Int J Hyperthermia, 2021, 38(1): 55-64.

[11]Monpeyssen H, Ben Hamou A, Hegedüs L, et al. High-intensity focused ultrasound (HIFU) therapy for benign thyroid nodules: a 3-year retrospective multicenter follow-up study[J]. Int J Hyperthermia. 2020, 37(1): 1301-1309.

[12]Ben Hamou A, Ghanassia E, Espiard S, et al. Safety and efficacy of thermal ablation (radiofrequency and laser): should we treat all types of thyroid nodules?[J]. Int J Hyperthermia. 2019, 36(1): 666-676.

[13]Russell JO, Desai DD, Noel JE, et al. Radiofrequency ablation of benign thyroid nodules: A prospective, multi-institutional North American experience[J]. Surgery. 2024, 175(1): 139-145.

[14]Akgun E, Romero-Velez G, Berber E. Assessing the efficacy of thyroid nodule radiofrequency ablation using patient-reported outcome measures[J]. Surgery. 2024, 175(3): 654-660.

[15]Kim GS, Seeley H, Noel J, et al. The safety and efficacy of radiofrequency ablation in benign pediatric thyroid disease in the US: An initial case series[J]. Laryngoscope Investig Otolaryngol. 2024 , 9(1): e1198.

[16]Ahmad S, Aljammal J, Orozco I, et al. Radiofrequency Ablation of Cervical Thyroid Cancer Metastases-Experience of Endocrinology Practices in the United States[J]. J Endocr Soc. 2023, 7(7): 066.

[17]Hussein M, Toraih E, Issa PP, et al. From ablation to operation: Unraveling the surgical outcomes and complications of thyroidectomy after radiofrequency ablation[J]. Surgery. 2024, 175(1): 146-152.

[18]Kandil E, Omar M, Aboueisha M, et al. Efficacy and Safety of Radiofrequency Ablation of Thyroid Nodules: A Multi-institutional Prospective Cohort Study[J]. Ann Surg. 2022, 276(4): 589-596.

[19]Kim J, Sun Z, Cummins M, et al. Implications of radiofrequency ablation in patients undergoing thyroid surgery for benign disease in the United States[J]. Surgery. 2022, 171(1): 160-164.

[20]Hussain I, Zulfiqar F, Li X, et al. Safety and Efficacy of Radiofrequency Ablation of Thyroid Nodules-Expanding Treatment Options in the United States[J]. J Endocr Soc. 2021, 5(8): 110.

[21]Hamidi O, Callstrom MR, Lee RA, et al. Outcomes of Radiofrequency Ablation Therapy for Large Benign Thyroid Nodules: A Mayo Clinic Case Series[J]. Mayo Clin Proc. 2018 , 93(8): 1018-1025.

[22]Dobnig H, Amrein K. Monopolar Radiofrequency Ablation of Thyroid Nodules: A Prospective Austrian Single-Center Study[J]. Thyroid. 2018, 28(4): 472-480.

[23]Nguyen VB, Nguyen Thi X, Nguyen Van Vy H, et al. Radiofrequency versus Ethanol Ablation for Single-Session Treatment of Benign Cystic Thyroid Nodules: A Short-Term Retrospective Study[J]. Ther Clin Risk Manag. 2023, 19: 97-104.

[24]Vu DL, Pham MT, Nguyen VB, et al. Efficacy and Safety of Radiofrequency Ablation for the Treatment of Autonomously Functioning Thyroid Nodules: A Long-Term Prospective Study[J]. Ther Clin Risk Manag. 2022, 18:11-19.

[25]Nguyen V B, Nguyen T X, Nguyen V V H, et al. Efficacy and Safety of Single‐Session Radiofrequency Ablation in Treating Benign Thyroid Nodules: A Short‐Term Prospective Cohort Study[J]. International Journal of Endocrinology, 2021, 2021(1): 7556393.

[26]Khanh HQ, Hung NQ, Vinh VH, et al. Efficacy of Microwave Ablation in the Treatment of Large (≥3 cm) Benign Thyroid Nodules[J]. World J Surg. 2020, 44(7): 2272-2279.

[27]Vuong NL, Dinh LQ, Bang HT, et al. Radiofrequency Ablation for Benign Thyroid Nodules: 1-Year Follow-Up in 184 Patients[J]. World J Surg. 2019, 43(10):2447-2453.

[28]Kovatcheva RD, Vlahov JD, Stoinov JI, et al. Benign Solid Thyroid Nodules: US-guided High-Intensity Focused Ultrasound Ablation-Initial Clinical Outcomes[J]. Radiology. 2015 , 276(2): 597-605.

[29]Prakash PS, Oh HB, Tan WB, et al. The Efficacy and Safety of High-Intensity Focused Ultrasound (HIFU) Therapy for Benign Thyroid Nodules-A Single Center Experience from Singapore[J]. World J Surg. 2019, 43(8): 1957-1963.

[30]Bom WJ, Joosten FBM, van Borren MMGJ, et al. Radiofrequency ablation for symptomatic, non-functioning, thyroid nodules: a single-center learning curve[J]. Endocr Connect. 2022, 11(1):e210304.

[31]de Boer H, Bom W, Veendrick P, et al. Hyperactive thyroid nodules treated by radiofrequency ablation: a Dutch single-centre experience[J]. Neth J Med. 2020, 78(2): 64-70.

[32]Yildirim G, Karakas HM. Uncooled Microwave Ablation as a Treatment Option to Preserve Thyroid Function in Patients with Benign Thyroid Nodules[J]. J Belg Soc Radiol. 2022, 106(1): 50.

[33]Aysan E, Idiz UO, Akbulut H, Elmas L. Single-session radiofrequency ablation on benign thyroid nodules: a prospective single center study : Radiofrequency ablation on thyroid[J]. Langenbecks Arch Surg. 2016, 401(3): 357-63.

[34]Ugurlu MU, Uprak K, Akpinar IN, et al. Radiofrequency ablation of benign symptomatic thyroid nodules: prospective safety and efficacy study[J]. World J Surg. 2015, 39(4): 961-8.

[35]Eisele RM, Scherber PR, Schlüter M, et al. Thermoablation of thyroid nodules reveals excellent results with low morbidity[J]. Technol Health Care. 2022, 30(3): 683-689.

[36]Vorländer C, Fischer A, Korkusuz H. High intensity focused ultrasound in the therapy of benign thyroid nodules-first German bicentric study with long-term follow-up[J]. Endocrine. 2022, 77(1): 112-120.

[37]Fischer A, Korkusuz H, Vorländer C. Effectiveness of High-intensity Focused Ultrasound (HIFU) Therapy of Solid and Complex Benign Thyroid Nodules - A Long-term Follow up Two-center Study[J]. Exp Clin Endocrinol Diabetes. 2022, 130(6): 374-380.

[38]Korkusuz Y, Mader OM, Kromen W, et al. Cooled microwave ablation of thyroid nodules: Initial experience[J]. Eur J Radiol. 2016, 85(11): 2127-2132.

[39]Crespo Vallejo E, Hermosin A, Gargallo M, et al. Multiple overlapping microwave ablation in benign thyroid nodule: a single-center 24-month study[J]. Eur Thyroid J. 2023, 12(1): e220175.

[40]Sambo Salas ME, Añez Ramos RJ, López Guerra A, et al. Morphological, clinical, and functional efficacy in the short and medium-term after radiofrequency treatment of predominantly solid, large, and clinically relevant thyroid nodules in patients who are not candidates for surgery: The experience after 100 procedures[J]. Endocrinol Diabetes Nutr (Engl Ed). 2022, 69(10): 816-827.

[41]Rodriguez Escobedo R, Martinez Tames G, Lanes Iglesias S, et al. Efficacy in size and symptom reduction of radiofrequency ablation of benign non-functioning thyroid nodules[J]. Endocrinol Diabetes Nutr (Engl Ed). 2022, 69(3): 194-200.

[42]Squarcia M, Mora M, Aranda G, Carrero E, et al. Long-Term Follow-Up of Single-Fiber Multiple Low-Intensity Energy Laser Ablation Technique of Benign Thyroid Nodules[J]. Front Oncol. 2021, 11:584265.

[43]Familiar Casado C, Merino Menendez S, Ganado Diaz T, et al. Single-session treatment of benign thyroid nodules with radiofrequency ablation: Results at 6 months in 24 patients[J]. Endocrinol Diabetes Nutr (Engl Ed). 2020, 67(3): 164-171.

[44]Aldea Martínez J, Aldea Viana L, López Martínez JL, et al. Radiofrequency Ablation of Thyroid Nodules: A Long-Term Prospective Study of 24 Patients[J]. J Vasc Interv Radiol. 2019 , 30(10): 1567-1573.

[45]Mauri G, Papini E, Bernardi S, et al. Image-guided thermal ablation in autonomously functioning thyroid nodules. A retrospective multicenter three-year follow-up study from the Italian Minimally Invasive Treatment of the Thyroid (MITT) Group[J]. Eur Radiol. 2022, 32(3): 1738-1746.

[46]Orlandi D, Viglino U, Dedone G, et al. US-CT fusion-guided percutaneous radiofrequency ablation of large substernal benign thyroid nodules[J]. Int J Hyperthermia. 2022, 39(1): 847-854.

[47]Gambelunghe G, Ristagno S, Stefanetti E,et al. Ultrasound-guided laser ablation of very large benign thyroid nodules: 4-year, retrospective follow-up in 24 patients[J]. Int J Hyperthermia. 2022, 39(1): 217-221.

[48]Cesareo R, Manfrini S, Pasqualini V, et al. Laser Ablation Versus Radiofrequency Ablation for Thyroid Nodules: 12-Month Results of a Randomized Trial (LARA II Study)[J]. J Clin Endocrinol Metab. 2021, 106(6): 1692-1701.

[49]Gambelunghe G, Stefanetti E, Avenia N, et al. Percutaneous Ultrasound-Guided Laser Ablation of Benign Thyroid Nodules: Results of 10-Year Follow-Up in 171 Patients[J]. J Endocr Soc. 2021, 5(7):081.

[50]Maurilio Deandrea, Francesca Garino, Mormile Alberto, et al. Radiofrequency ablation for benign thyroid nodules according to different ultrasound features: an Italian multicentre prospective study[J]. European Journal of Endocrinology. 2019,180(1): 79–87.

[51]Cappelli C, Franco F, Pirola I, et al. Radiofrequency ablation of functioning and non-functioning thyroid nodules: a single institution 12-month survey[J]. J Endocrinol Invest. 2020, 43(4): 477-482.

[52]Rabuffi P, Spada A, Bosco D, et al. Treatment of thyroid nodules with radiofrequency: a 1-year follow-up experience[J]. J Ultrasound. 2019, 22(2): 193-199.

[53]Oddo S, Felix E, Mussap M, et al. Quality of Life in Patients Treated with Percutaneous Laser Ablation for Non-Functioning Benign Thyroid Nodules: A Prospective Single-Center Study[J]. Korean J Radiol. 2018, 19(1): 175-184.

[54]Pacella CM, Mauri G, Cesareo R, et al. A comparison of laser with radiofrequency ablation for the treatment of benign thyroid nodules: a propensity score matching analysis[J]. Int J Hyperthermia. 2017, 33(8):911-919.

[55]Achille G, Zizzi S, Di Stasio E,et al. Ultrasound-guided percutaneous laser ablation in treating symptomatic solid benign thyroid nodules: Our experience in 45 patients[J]. Head Neck. 2016 , 38(5): 677-82.

[56]Pacella CM, Mauri G, Achille G,et al. Outcomes and Risk Factors for Complications of Laser Ablation for Thyroid Nodules: A Multicenter Study on 1531 Patients[J]. J Clin Endocrinol Metab. 2015, 100(10): 3903-10.

[57]Bernardi S, Dobrinja C, Fabris B, et al. Radiofrequency ablation compared to surgery for the treatment of benign thyroid nodules[J]. International Journal of Endocrinology, 2014, 2014(1): 934595.

[58]Pacella CM, Bizzarri G, Spiezia S, et al. Thyroid tissue: US-guided percutaneous laser thermal ablation. Radiology[J]. 2004, 232(1): 272-80.

[59]Chiang PL, Luo SD, Chang YH, et al. Radiofrequency ablation for thyroid Bethesda III nodules: preliminary results[J]. Eur Thyroid J. 2023, 12(6): e230105.

[60]Lin WC, Wang CK, Wang WH, et al. Multicenter Study of Benign Thyroid Nodules with Radiofrequency Ablation: Results of 762 Cases over 4 Years in Taiwan[J]. J Pers Med. 2022, 12(1): 63.

[61]Lin AN, Lin WC, Cheng KL, et al. Radiofrequency Ablation a Safe and Effective Treatment for Pediatric Benign Nodular Thyroid Goiter[J]. Front Pediatr. 2021, 9:753343.

[62]Lin WC, Kan NN, Chen HL, et al. Efficacy and safety of single-session radiofrequency ablation for benign thyroid nodules of different sizes: a retrospective study[J]. Int J Hyperthermia. 2020, 37(1): 1082-1089.

[63]Lang BHH, Fung MMH. Safety and Efficacy of Single-Session Radiofrequency Ablation Treatment for Benign Non-toxic Multinodular Goiter[J]. World J Surg. 2022, 46(7): 1704-1710.

[64]Guo R, Zheng B, Wu T, et al. The two-year prognosis of multinodular goiter following radiofrequency ablation: based on all nodule burdens[J]. Eur Thyroid J. 2024, 13(1): e230134.

[65]Zhang Y, Han X, Ren YJ, et al Microwave ablation versus radiofrequency ablation for solid or predominantly solid benign thyroid nodules: a randomized controlled clinical trial[J]. Zhonghua Nei Ke Za Zhi. 2024, 63(1): 74-80.

[66]Lin Y, Wu ZR, Shi YP, et al. Radiofrequency Ablation of Unifocal Papillary Thyroid Microcarcinoma With BRAF V600E Mutation[J]. J Clin Endocrinol Metab. 2023, 108(11): e1298-e1305.

[67]Lou Q, Zhu YF, Ye ML. Treatment of Cystic-Solid Thyroid Nodules with Ultrasound-Guided Radiofrequency Ablation and Enhancement of Thyroid Function[J]. J Multidiscip Healthc. 2023, 16: 2773-2779.

[68]Min X, Zhang Z, Chen Y, et al. Comparison of the effectiveness of lauromacrogol injection for ablation and microwave ablation in the treatment of predominantly cystic thyroid nodules: a multicentre study[J]. BMC Cancer. 2023, 23(1): 785.

[69]Yan L, Li XY, Li Y, et al. Ultrasound-Guided Radiofrequency Ablation versus Thyroidectomy for the Treatment of Benign Thyroid Nodules in Elderly Patients: A Propensity-Matched Cohort Study[J]. AJNR Am J Neuroradiol. 2023, 44(6): 693-699.

[70]Juan Z, Yongping L, Han X, et al. A 5-year follow-up study on the efficacy and safety of ultrasound-guided laser ablation in elderly patients with papillary thyroid microcarcinoma: A retrospective, single-center study from China[J]. Front Endocrinol (Lausanne). 2022, 13: 972589.

[71]Du JR, Li WH, Quan CH, et al. Long-term outcome of microwave ablation for benign thyroid nodules: Over 48-month follow-up study[J]. Front Endocrinol (Lausanne). 2022, 13:941137.

[72]Lin Y, Shi YP, Tang XY, et al. Significance of radiofrequency ablation in large solid benign thyroid nodules[J]. Front Endocrinol (Lausanne). 2022, 13: 902484.

[73]Li L, Qiu X. Safety and Efficacy of Ultrasound-Guided Radiofrequency Ablation for Benign Nonfunctional Thyroid Nodules in Children: A Retrospective Study of 62 Patients with Over Four Years of Follow-Up[J]. Thyroid. 2022, 32(5): 525-535.

[74]Li S, Yang M, Guo H, Liu M, et al. Microwave Ablation Vs Traditional Thyroidectomy for Benign Thyroid Nodules: A Prospective, Non-Randomized Cohort Study[J]. Acad Radiol. 2022 , 29(6): 871-879.

[75]Yang J, Zhang Y, Li X, et al. Efficacy and safety of ultrasound-guided microwave ablation versus surgical resection for Bethesda category IV thyroid nodules: A retrospective comparative study[J]. Front Endocrinol (Lausanne). 2022, 13:924993.

[76]Li Y, He H, Li W, Zhao J, et al. Efficacy and safety of radiofrequency ablation for calcified benign thyroid nodules: results of over 5 years' follow-up[J]. BMC Med Imaging. 2022, 22(1): 75.

[77]Wu J, Wei Y, Zhao ZL, et al. A preliminary study of microwave ablation for solitary T1N0M0 papillary thyroid carcinoma with capsular invasion[J]. Int J Hyperthermia. 2022, 39(1):372-378.

[78]Peng K, Zhou P, Liu W. Long-Term Efficacy of Ultrasound-Guided Percutaneous Laser Ablation for Low-Risk Papillary Thyroid Microcarcinoma: A 5-Year Follow-Up Study[J]. Biomed Res Int. 2021 , 2021:6616826.

[79]Honglei G, Shahbaz M, Farhaj Z, et al. Ultrasound guided microwave ablation of thyroid nodular goiter and cystadenoma: A single center, large cohort study[J]. Medicine (Baltimore). 2021, 100(34): e26943.

[80]Wu J, Zhao ZL, Cao XJ, et al. A feasibility study of microwave ablation for papillary thyroid cancer close to the thyroid capsule[J]. Int J Hyperthermia. 2021, 38(1):1217-1224.

[81]Xia B, Yu B, Wang X, et al. Conspicuousness and recurrence related factors of ultrasound-guided microwave ablation in the treatment of benign thyroid nodules[J]. BMC Surg. 2021, 21(1):317.

[82]Jin H, Fan J, Lu L,et al. A Propensity Score Matching Study Between Microwave Ablation and Radiofrequency Ablation in Terms of Safety and Efficacy for Benign Thyroid Nodules Treatment[J]. Front Endocrinol (Lausanne). 2021, 12:584972.

[83]Yang H, Chen Y, Chen B, et al. Ablating Aspiration Needle Tract Prior to Microwave Ablation Can Improve Therapeutic Outcomes for Predominantly Cystic Thyroid Nodules[J]. Front Endocrinol (Lausanne). 2021, 12:752822.

[84]Yan L, Zhang M, Xie F, et al. Efficacy and safety of radiofrequency ablation for benign thyroid nodules in patients with previous thyroid lobectomy[J]. BMC Med Imaging. 2021, 21(1):47.

[85]Yan L, Deng C, Song Q, et al. Radiofrequency ablation versus reoperation for benign thyroid nodules that developed after previous thyroid surgery[J]. Int J Hyperthermia. 2021, 38(1):176-182.

[86]Li X, Lan Y, Li N,et al. Ultrasound-Guided Thermal Ablation of Bethesda IV Thyroid Nodules: A Pilot Study[J]. Front Endocrinol (Lausanne). 2021, 12:674970.

[87]Zhao J, Qian L, Liu Y, Tan X. A long-term retrospective study of ultrasound-guided microwave ablation of thyroid benign solid nodules. Int J Hyperthermia. 2021;38(1):1566-1570. doi: 10.1080/02656736.2021.1994659. PMID: 34727816.

[88]Fu QQ, Kang S, Wu CP, et al. A study on the efficacy of microwave ablation for benign thyroid nodules and related influencing factors[J]. Int J Hyperthermia. 2021, 38(1):1469-1475.

[89]Cai W, Liu S, Yu X, et al Is partial ablation appropriate for benign thyroid nodules? A retrospective study with long-term follow-up after microwave ablation[J]. Int J Hyperthermia. 2021, 38(1):923-930.

[90]He H, Song Q, Lan Y, et al. Efficacy and safety of ultrasound-guided radiofrequency ablation for low-risk papillary thyroid microcarcinoma in patients aged 55 years or older: a retrospective study[J]. Int J Hyperthermia. 2021, 38(1):604-610.

[91]Wang X, Niu X, Mu S, et al. Analysis and evaluation of the efficacy of ultrasound-guided microwave ablation for papillary thyroid microcarcinoma[J]. Int J Hyperthermia. 2021, 38(1):1476-1485.

[92]Hu K, Lian Y, Wang J, et al. Management of bleeding associated with radiofrequency ablation of benign thyroid nodules[J]. J Int Med Res. 2020, 48(8): 300060520937526.

[93]Guo Y, Li Z, Wang S, et al. Single-Fiber Laser Ablation in Treating Selected Metastatic Lymph Nodes of Papillary Thyroid Carcinoma and Benign Cold Thyroid Nodules-Preliminary Results[J]. Lasers Surg Med. 2020, 52(5):408-418.

[94]Yao Z, Wu T, Zheng B, et al. A Novel Strategy for Single-Session Ultrasound-Guided Radiofrequency Ablation of Large Benign Thyroid Nodules: A Pilot Cohort Study[J]. Front Endocrinol (Lausanne). 2020, 11:560508.

[95]Zhang M, Tufano RP, Russell JO,et al. Ultrasound-Guided Radiofrequency Ablation Versus Surgery for Low-Risk Papillary Thyroid Microcarcinoma: Results of Over 5 Years' Follow-Up[J]. Thyroid. 2020, 30(3):408-417.

[96]Wu R, Luo Y, Tang J, et al. Ultrasound-guided radiofrequency ablation for papillary thyroid microcarcinoma: a retrospective analysis of 198 patients[J]. Int J Hyperthermia. 2020, 37(1):168-174.

[97]Guang Y, He W, Luo Y, et al. Patient satisfaction of radiofrequency ablation for symptomatic benign solid thyroid nodules: our experience for 2-year follow up[J]. BMC Cancer. 2019, 19(1):147.

[98]Ji L, Wu Q, Gu J, Deng X, et al. Ultrasound-guided percutaneous laser ablation for papillary thyroid microcarcinoma: a retrospective analysis of 37 patients[J]. Cancer Imaging. 2019, 19(1):16.

[99]Teng DK, Li HQ, Sui GQ, et al. Preliminary report of microwave ablation for the primary papillary thyroid microcarcinoma: a large-cohort of 185 patients feasibility study[J]. Endocrine. 2019 , 64(1):109-117.

[100]Cui R, Yu J, Han ZY, et al. Ultrasound-Guided Percutaneous Microwave Ablation for Substernal Goiter: Initial Experience[J]. J Ultrasound Med. 2019, 38(11):2883-2891.

[101]Jin H, Fan J, Liao K, et al. A propensity score matching study between ultrasound-guided percutaneous microwave ablation and conventional thyroidectomy for benign thyroid nodules treatment[J]. Int J Hyperthermia. 2018, 35(1):232-238.

[102]Tang X, Cui D, Chi J, et al. Evaluation of the safety and efficacy of radiofrequency ablation for treating benign thyroid nodules[J]. J Cancer. 2017, 8(5):754-760.

[103]Wu W, Gong X, Zhou Q, et al. Ultrasound-Guided Percutaneous Microwave Ablation for Solid Benign Thyroid Nodules: Comparison of MWA versus Control Group[J]. Int J Endocrinol. 2017, 2017:9724090.

[104]Ding M, Tang X, Cui D, et al. Clinical outcomes of ultrasound-guided radiofrequency ablation for the treatment of primary papillary thyroid microcarcinoma[J]. Clin Radiol. 2019 , 74(9):712-717.

[105]Zhang M, Luo Y, Zhang Y, et al. Efficacy and Safety of Ultrasound-Guided Radiofrequency Ablation for Treating Low-Risk Papillary Thyroid Microcarcinoma: A Prospective Study[J]. Thyroid. 2016, 26(11):1581-1587.

[106]Ma S, Zhou P, Wu X, et al. Detection of the Single-Session Complete Ablation Rate by Contrast-Enhanced Ultrasound during Ultrasound-Guided Laser Ablation for Benign Thyroid Nodules: A Prospective Study.[J] Biomed Res Int. 2016; 2016:9565364.

[107]Liu C, Wu B, Huang P, et al. US-Guided Percutaneous Microwave Ablation for Primary Hyperparathyroidism with Parathyroid Nodules: Feasibility and Safety Study[J]. J Vasc Interv Radiol. 2016, 27(6):867-75.

[108]Che Y, Jin S, Shi C, et al. Treatment of Benign Thyroid Nodules: Comparison of Surgery with Radiofrequency Ablation[J]. AJNR Am J Neuroradiol. 2015, 36(7):1321-5.

[109]Yue W, Wang S, Yu S, et al. Ultrasound-guided percutaneous microwave ablation of solitary T1N0M0 papillary thyroid microcarcinoma: initial experience[J]. Int J Hyperthermia. 2014, 30(2):150-157.

[110]Feng B, Liang P, Cheng Z, et al. Ultrasound-guided percutaneous microwave ablation of benign thyroid nodules: experimental and clinical studies[J]. Eur J Endocrinol. 2012, 166(6):1031-1037.

# Supplementary Tables

Table1. The comparison of complication morbidity between SHNH and China

| Complications | SHNH  n=536 (%) | China  n=5481 (%) | *P* |
| --- | --- | --- | --- |
| Vasovagal reaction | 5(1.05) | 1(0.02) | ＜0.0001* |
| Dyspnea | 2(0.42) | 0(0.00) | 0.0080* |
| Scar | 2(0.42) | 0(0.00) | 0.0080* |
| Infection | 2(0.42) | 0(0.00) | 0.0080* |
| Voice change | 6(1.26) | 106(2.18) | 0.1770 |
| Nodule rupture | 0(0.00) | 18(0.37) | 0.3988 |
| Horner’s syndrome | 1(0.21) | 2(0.04) | 0.2456 |
| Other.nerve.injury | 0(0.00) | 0(0.00) | 1.0000 |
| Tracheal injury | 1(0.21) | 0(0.00) | 0.0896 |
| Esophageal injury | 0(0.00) | 1(0.02) | 1.0000 |
| Postoperative hyperthyroidism | 0(0.00) | 2(0.04) | 1.0000 |
| Postoperative hypothyroidism | 0(0.00) | 20(0.41) | 0.2501 |
| Postoperative Hashimoto thyroiditis | 0(0.00) | 0(0.00) | 1.0000 |
| Dysphagia | 0(0.00) | 1(0.02) | 1.0000 |
| Hematoma | 5(1.05) | 34(0.70) | 0.3919 |
| Skin burn | 2(0.42) | 11(0.23) | 0.7448 |
| Fever | 0(0.00) | 8(0.16) | 1.0000 |
| Pain | 25(5.23) | 333(6.86) | 0.1745 |
| Edema | 6(1.26) | 77(1.59) | 0.5772 |
| Nausea and vomiting | 0(0.00) | 8(0.16) | 1.0000 |
| Cough | 1(0.21) | 4(0.08) | 0.3748 |
| Total | 58(10.82) | 626(11.42) | 1.0000 |

* *P*<0.05 was statistically significant.

Table 2. The comparison of complication morbidity between SHNH and China

| Complications | SHNH  n=536 (%) | Other countries  n=14668 (%) | *P* |
| --- | --- | --- | --- |
| Tracheal injury | 1(0.21) | 0(0.00) | 0.0360* |
| Vasovagal reaction | 5(1.05) | 47(0.37) | 0.0383* |
| Dyspnea | 2(0.42) | 1(0.01) | 0.0038* |
| Scar | 2(0.42) | 2(0.02) | 0.0003* |
| Infection | 2(0.42) | 6(0.05) | 0.0213* |
| Fever | 0(0.00) | 169(1.32) | 0.0031* |
| Voice change | 6(1.26) | 230(1.57) | 0.3860 |
| Nodule rupture | 0(0.00) | 36(0.25) | 0.6399 |
| Horner’s syndrome | 1(0.21) | 4(0.03) | 0.1667 |
| Other nerve injury | 0(0.00) | 5(0.03) | 1.0000 |
| Esophageal injury | 0(0.00) | 1(0.01) | 1.0000 |
| Postoperative hyperthyroidism | 0(0.00) | 20(0.14) | 1.0000 |
| Postoperative hypothyroidism | 0(0.00) | 49(0.33) | 0.4229 |
| Postoperative Hashimoto thyroiditis | 0(0.00) | 4(0.03) | 1.0000 |
| Dysphagia | 0(0.00) | 12(0.08) | 1.0000 |
| Hematoma | 5(1.05) | 169(1.15) | 0.8363 |
| Skin burn | 2(0.42) | 28(0.19) | 0.6756 |
| Pain | 25(5.23) | 836(5.70) | 0.2684 |
| Edema | 6(1.26) | 186(1.27) | 0.7316 |
| Nausea and vomiting | 0(0.00) | 23(0.16) | 1.0000 |
| Cough | 1(0.21) | 22(0.15) | 0.5681 |
| Total | 58(10.82) | 1850(12.61) | 1.0000 |

* *P*<0.05 was statistically significant.
